# Creatine and cyclocreatine treatment of human colon adenocarcinoma xenografts: ^31^P and ^1^H magnetic resonance spectroscopic studies

**DOI:** 10.1038/sj.bjc.6690045

**Published:** 1999-01

**Authors:** C A Kristensen, N Askenasy, R K Jain, A P Koretsky

**Affiliations:** The Edwin L. Steele Laboratory, Department of Radiation Oncology, Massachusetts General Hospital, Harvard Medical School, Boston, USA; NMR Center, Carnegie-Mellon University, Pittsburgh, PA 15213, USA

**Keywords:** creatine, cyclocreatine, tumour xenograft, magnetic resonance spectroscopy, tumour growth

## Abstract

Creatine (Cr) and cyclocreatine (cyCr) have been shown to inhibit the growth of a variety of human and murine tumours. The purpose of this study was to evaluate the anti-tumour effect of these molecules in relation to drug accumulation, energy metabolism, tumour water accumulation and toxicity. Nude mice carrying a human colon adenocarcinoma (LS174T) with a creatine kinase (CK) activity of 2.12 units mg^−1^ protein were fed Cr (2.5% or 5%) or cyCr (0.025%, 0.1% or 0.5%) for 2 weeks and compared with controls fed standard diet. Cr concentrations of 2.5% and 5% significantly inhibited tumour growth, as did 0.1% and 0.5% cyCr. In vivo ^31^P magnetic resonance spectroscopy (MRS) after 2 weeks of treatment showed an increase in [phosphocreatine (PCr)+phosphocyclocreatine (PcyCr)]/nucleoside triphosphate (NTP) with increasing concentrations of dietary Cr and cyCr, without changes in absolute NTP contents. The antiproliferative effect of the substrates of CK was not related to energy deficiency but was associated with acidosis. Intratumoral substrate concentrations (measured by ^1^H-MRS) of 4.8 μmol g^−1^ wet weight Cr (mice fed 2.5% Cr) and 6.2 μmol g^−1^ cyCr (mice fed 0.1% cyCr) induced a similar decrease in growth rate, indicating that both substrates were equally potent in tumour growth inhibition. The best correlant of growth inhibition was the total Cr or (cyCr+Cr) concentrations in the tissue. In vivo, these agents did not induce excessive water accumulation and had no systemic effects on the mice (weight loss, hypoglycaemia) that may have caused growth inhibition. © 1999 Cancer Research Campaign


*British Journal of Cancer* (1999) **79**(2), 278–285
© 1999 Cancer Research Campaign

### Creatine and cyclocreatine treatment of human colon adenocarcinoma xenografts: $^{31}P$ and $^{1}H$ magnetic resonance spectroscopic studies

**CA Kristensen[1], N Askenasy[2], RK Jain[1] and AP Koretsky[2]**

[1]The Edwin L. Steele Laboratory, Department of Radiation Oncology, Massachusetts General Hospital, Harvard Medical School, Boston, MA 02114;
[2]NMR Center, Carnegie-Mellon University, Pittsburgh, PA 15213, USA

**Summary** Creatine (Cr) and cyclocreatine (cyCr) have been shown to inhibit the growth of a variety of human and murine tumours. The purpose of this study was to evaluate the anti-tumour effect of these molecules in relation to drug accumulation, energy metabolism, tumour water accumulation and toxicity. Nude mice carrying a human colon adenocarcinoma (LS174T) with a creatine kinase (CK) activity of 2.12 units $mg^{-1}$ protein were fed Cr (2.5% or 5%) or cyCr (0.025%, 0.1% or 0.5%) for 2 weeks and compared with controls fed standard diet. Cr concentrations of 2.5% and 5% significantly inhibited tumour growth, as did 0.1% and 0.5% cyCr. In vivo $^{31}P$ magnetic resonance spectroscopy (MRS) after 2 weeks of treatment showed an increase in [phosphocreatine (PCr)+phosphocyclocreatine (PcyCr)]/nucleoside triphosphate (NTP) with increasing concentrations of dietary Cr and cyCr, without changes in absolute NTP contents. The antiproliferative effect of the substrates of CK was not related to energy deficiency but was associated with acidosis. Intratumoral substrate concentrations (measured by $^{1}H$-MRS) of 4.8 μmol $g^{-1}$ wet weight Cr (mice fed 2.5% Cr) and 6.2 μmol $g^{-1}$ cyCr (mice fed 0.1% cyCr) induced a similar decrease in growth rate, indicating that both substrates were equally potent in tumour growth inhibition. The best correlant of growth inhibition was the total Cr or (cyCr+Cr) concentrations in the tissue. In vivo, these agents did not induce excessive water accumulation and had no systemic effects on the mice (weight loss, hypoglycaemia) that may have caused growth inhibition.

**Keywords:** creatine; cyclocreatine; tumour xenograft; magnetic resonance spectroscopy; tumour growth

Creatine (Cr) and its analogue, 2-imino-1-imidazolidineacetic acid or cyclocreatine (cyCr), have both been demonstrated to have anti-tumour effect in vivo in some, but not all, transplanted human and rodent tumours (Lillie et al, 1993; Miller et al, 1993). Cyclocreatine also has an anti-tumour effect in vitro (Martin et al, 1994*a*, *b*; Schiffenbauer et al, 1995, 1996; Bergnes et al, 1996) and has been shown to induce intracellular water accumulation (Schiffenbauer et al, 1995). Furthermore, cyCr inhibits tumour cell proliferation in all phases of the cell cycle (Martin et al, 1994*b*) and induces stabilization of microtubules (Martin et al, 1995). The phosphorylation of cyCr by creatine kinase (CK) (Cr + MgATP → PCr + MgADP + $H^{+}$) seems to be crucial for the anti-tumour effect because (1) the growth-inhibitory effect of cyCr is dependent on in vitro CK activity and (2) transfection of the creatine kinase gene into cells with low CK activity increases the in vitro growth-inhibitory effect of cyCr significantly (Lillie et al, 1993). In a recently published study of two different tumour cell lines, cellular swelling and phosphocyclocreatine (PcyCr) accumulation was observed only in the cell line with the highest creatine kinase activity, in spite of growth inhibition of both cell lines (Schiffenbauer et al, 1996).

CK activity has been associated with tumour growth in several studies, but a direct relationship has not been derived. CK is overexpressed in most tumours compared with their non-malignant tissues of origin (Shatton et al, 1979), and growth inhibition of breast cancer tumours by oestrogen withdrawal was shown to down-regulate creatine kinase activity (Kristensen et al, 1995). Elevated plasma creatine kinase activity is a poor prognostic factor in several cancers, e.g. small-cell lung cancer (Carney et al, 1984), possibly as a result of leakage of enzymes from tumour tissue to plasma after, for example, myocardial infarcts. Despite the lack of sufficient information on the nature and intracellular localization of the CK isoenzymes in different tumours, it is apparent that the isoforms have distinct roles in neoplastic tissues. Plasma levels of the cytosolic brain isoenzyme (CK-B) were found to correlate with the aggressiveness of the neoplastic clone (Neri et al, 1985). The oncogenic activity of adenovirus E1a was accompanied by induction of CK-B expression (Kaddurah-Daouk et al, 1990), while the tumour-suppressor activity of p53 was associated with a positive regulation of the cytosolic muscle isoenzyme (CK-M) and a negative regulation of CK-B (Zhao et al, 1994).

CK isoenzymes have also been associated with tissue growth and development in non-malignant tissues. During developmental stages, fetal tissues express mainly CK-B (Hall and DeLuca, 1976). The mitochondrial isoenzyme (CK-mit) is expressed during the post-natal period (McAuliffe et al, 1989) and is associated with the development of the oxidative capacity of the tissue (Perry et al, 1988; Walliman et al, 1992). Recently, it has been demonstrated that the specific CK isoform expression may be crucial in achieving selective inhibitory effects of Cr and cyCr on liver regeneration (Askenasy and Koretsky, 1997).

The aim of the present study was to determine the growth-inhibitory effects of Cr and cyCr on LS174T human colon

*Received 6 November 1997*
*Revised 25 April 1998*
*Accepted 30 April 1998*

*Correspondence to:* CA Kristensen, Institute of Molecular Pathology, University of Copenhagen, Frederik d. V's Vej 11, 5., DK-2100 Copenhagen, Denmark adenocarcinoma implanted subdermally in nude mice. This tumour line has previously been used for extensive studies of tumour physiology and drug therapy, it has a relatively high CK activity and it is growth inhibited by Cr and cyCr. In view of the mechanisms presumed to contribute to the growth-inhibitory effects of these agents, we have evaluated: (1) intratumoral accumulation of Cr and cyCr delivered orally; (2) concentrations of the phosphorylated products of the CK substrates (PCr and PcyCr) and energy metabolic state of the tumours determined by in vivo $^{31}$P-MRS; (3) tumour water content and (4) systemic toxicity of these agents during a 2-week feeding period. These data were analysed with reference to CK activity and the isoenzyme distribution in the tumour.

### MATERIALS AND METHODS

### Animals and tumour lines

LS174T is a human colon adenocarcinoma cell line (ATCC, Rockville, MD, USA) maintained in our lab as a cell culture and serially passed in nude mice. Six- to eight-week-old males of nu/Sed origin were anaesthetized with ketamine (Ketalar, Parke-Davis, Morris Plains, NJ, USA) 100 mg $kg^{-1}$ body weight, and xylazine (Rompun, Miles Inc, Shawnee Mission, KA, USA) 10 mg $kg^{-1}$ body weight, and 1-$mm^3$ LS174T tumour chunks were implanted subcutaneously in the right hind leg. Institutional guidelines for animal welfare and experimental conduct were followed.

### Experimental set-up

When the tumours became visible, the mice were split into a control group and six experimental groups; controls were fed standard liquid rat diet (LiquiDiet '82, Bioserv, Frenchtown, NJ, USA) from special glass liquid diet dispensers. The experimental groups were fed the same liquid diet mixed with different concentrations of Cr (Sigma Chemical, St Louis, MO, USA) and cyCr (Aldrich Chemical, Milwaukee, WI, USA) as follows: 2.5% Cr, 5% Cr, 0.025% cyCr, 0.1% cyCr, 0.5% cyCr and 1% cyCr (all concentrations are w/v). Diet and water were given ad libitum. For 2 weeks the tumours were measured bidimensionally three times weekly. The animals were weighed before and during the feeding, and the volume of diet consumed was read from the diet dispenser. At the end of the 2-week feeding period the animals were anaesthetized with ketamine/xylazine and in vivo $^{31}$P-MRS was performed. After spectroscopy, blood samples were taken for determination of serum glucose; tumour and liver samples were taken for determination of total concentrations of CK substrates (Cr + cyCr), lactate and alanine, wet weight–dry weight ratios and CK activity.

Tumour growth was measured in a similar set-up during treatment of each mouse with i.p. injections of 10 mg cyCr in 0.3 ml of saline daily and compared with growth of control tumours given i.p. saline injections. cyCr was dissolved in hot saline (60–70°C) in a water bath and cooled to 37°C before injection.

### Tumour growth curves

Mean tumour growth curves were constructed according to a transformed Gompertz function, as described by Spang-Thomsen et al (1980). For determination of the α-value, a simple transformation of the Gompertz function was used:

$$\text{Transformed tumour size} = \ln[\ln A(\max) - \ln A(t)] = \ln \beta/\alpha - \alpha t$$

where $\alpha$ and $\beta$ are constants, $A$(max) is the theoretical maximum size and $A(t)$ is the tumour size at time $t$. This growth function depicts a straight line with negative slope ($-\alpha$) when the transformed tumour size is plotted against time. ln $A$(max) was set to 7, corresponding to tumour dimensions of approximately $15 \times 15$ $mm^2$. Consequently, all variation in growth is placed in one single parameter, $\alpha$, that expresses the growth of the individual tumour.

### $^{31}$P-MRS

Spectra were collected with a 300-MHz Bruker Biospec spectrometer (horizontal wide bore) operating at 121.5 MHz for $^{31}$P. An 8-mm, two-turn surface coil was located above the tumour. Spectra were collections of 256 transients of 60° pulses at a repetition time of 1 s. Fully relaxed spectra were recorded at a repetition time of 10 s for calculation of the saturation factors for each individual phosphorus resonance ($n$ = 10). Free induction decays were multiplied with a Lorentzian window of 30 Hz. Peak areas were integrated with a Bruker software subroutine, after baseline correction. Phosphorus metabolites were quantified against an external reference containing 8 μmol of methylphosphonic acid (MPA) and 20 μmol of gadolinium diethylenetriaminopentacetic acid (gadolinium DTPA). Data are reported as μmol $coil^{-1}$ because the volume detected by the coil was not quantitated.

Saturation transfer experiments were performed to evaluate magnetization transfer from PCr to nucleoside triphosphate (NTP) (forward CK direction). $^{31}$P spectra were collections of 20 transients (90° pulses) with repetition times of 10 s. Magnetization transfer was measured using a narrow-band, low-power radiofrequency (RF) pulse with increasing durations (0.5, 1, 3, 5, 7 and 9 s). The saturating pulse was applied for each duration at the resonance frequency of γ-NTP ($M_z$) and at a symmetrical location downfield from the PCr peak ($M_o$). The apparent longitudinal relaxation time ($T_{lapp}$) was determined from the mono-exponential decay of $M_z/M_o$ (a pair of saturated/control spectra). Pseudo-first order rate constants ($k$) were calculated according to $k = (1 - M_{ss}/M_o/T_{lapp}$, where $M_{ss}$ is the steady-state value of $M_z$. The reaction velocity was calculated from the rate constant and the concentration of PCr (per surface coil detection volume).

### $^1$H-MRS

$^1$H-MRS was performed on perchloric acid extracts of tumour and liver tissue samples. Spectra were collected with a 300-MHz Bruker spectrometer (vertical narrow bore). Fully relaxed spectra were collections of eight transients of 90° pulses with a relaxation delay of 8 s. After baseline correction, peak areas were integrated with a Bruker software subroutine. Proton resonances were assigned against 1 μmol of trimethylsylilpropionic acid (TSP), which was also used to quantify the metabolites.

### Perchloric acid extracts

Tissue samples were diluted with 5 volumes of 5% perchloric acid, homogenized and centrifuged at 15 000 ***g*** for 15 min at 4°C. The supernatant was neutralized with potassium hydroxide and centrifuged again at 20 000 ***g*** for 20 min at 4°C. The supernatant was lyophilized and stored at –85°C. Extract powder was diluted in 0.6 ml of $D_2O$ in the presence of 1 mM ethyleneglycol (bis-aminoethyl ether)tetraacetic acid (EGTA) and 1 μM TSP.

© Cancer Research Campaign 1999

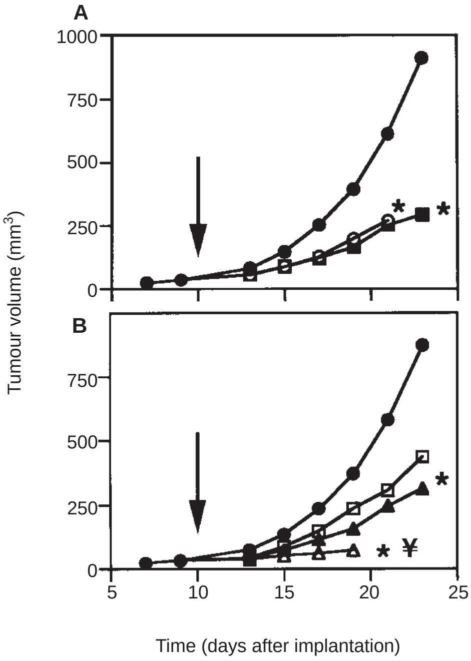

**Figure 1** Inhibitory effect of Cr (**A**) and cyCr (**B**) on growth of LS174T human colon adenocarcinomas. Cr in doses of 2.5% (○, *n* = 7) and 5% (■, *n* = 8) and cyclocreatine in doses of 0.025% (□, *n* = 8), 0.1% (▲, *n* = 6) and 0.5% (△, *n* = 4) were given in liquid diet from day 10 (arrow) after subcutaneous tumour implantation. The α-values for each experimental group were compared with those of control tumours (●, *n* = 7) by a *t*-test. **P* < 0.05. †All animals sacrificed on day 20 as a result of severe weight loss

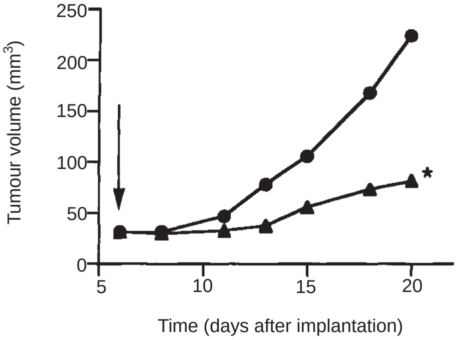

**Figure 2** Tumour growth-inhibitory effect of cyclocreatine 10 mg i.p. daily (▲, *n* = 9) compared with controls (●, *n* = 9). Treatment was initiated on day 6. **P* < 0.05

### CK activity

Tissue samples were homogenized in 99 parts of the following buffer: 26 mM Tris, 0.3 M sucrose, 20 mM β-mercaptoethanol and 1% *tert*.-octylphenoxy-poly(oxyethylene)ethanol (Sigma). CK activity was determined in the direction of Cr to PCr at 37°C by the spectrophotometric assay described previously (Kristensen et al, 1995) and in the opposite direction at 37°C by a commercially available kit (cat. no. 45-1, Sigma).

### Gel electrophoresis

CK distribution was tested in samples of tissue homogenate at a final dilution of 1:200. One-microlitre volumes were applied to a 1% agarose Multitrac CK electrophoretic isoenzyme gel (Ciba-Corning, Marshfield, MA, USA) which uses a hexokinase/glucose 6-phosphate dehydrogenase coupled enzyme system.

### Serum glucose

Serum glucose was determined spectrophotometrically using a commercially available kit (Sigma Diagnostics, St Louis, MO, USA, cat. no. 510-A).

### Tissue water content

Tissue samples were blotted and weighed (ww), and stored for 24 h at 85°C for determination of the dry weight (dw). Total tissue water content was calculated according to (ww–dw) $dw^{-1}$, and is expressed in units of ml $g^{-1}$ dw.

### Statistics

Data from each experimental group were compared with data from the control group by a *t*-test (SPSS statistics).

### RESULTS

### Tumour growth

The growth of control tumours and tumours treated with different doses of Cr and cyCr is shown in Figure 1. Both 2.5% and 5% Cr induced significant growth inhibition ($P < 0.05$) (Figure 1A), whereas the growth inhibition induced by the lowest dose of cyCr, 0.025%, was non-significant. Tumour growth was significantly inhibited by 0.1% and 0.5% cyCr ($P < 0.05$) (Figure 1B). Figure 2 shows the growth curve of tumours treated with i.p. injections of cyCr (10 mg daily) as compared with controls. Also i.p. injections of cyCr induced significant growth inhibition ($P < 0.05$).

### Toxicity

The animals in all five experimental groups fed Cr or cyCr lost significant amounts of weight during the feeding period compared with controls, and the weight loss was correlated to the mean amount of liquid diet intake ($r^2 = 0.82$). There was not, however, any correlation between weight loss and tumour growth inhibition measured as the α-value for each individual tumour ($r^2 = 0.16$). The mice fed 0.5% cyCr lost weight rapidly, probably because of the very limited diet intake, and all animals were sacrificed on day 20 because of severe (>30%) weight loss.

Mean serum glucose level after 2 weeks of feeding was 11.5 mM (range 3.2–24.9 mM) in the control group, 11.3 mM (range 6.5–16.2 mM) in the 2.5% Cr group, 14.2 mM (range 11.2–18.2 mM) in the 5% Cr group, 20.1 mM (range 13.2–23.2 mM) in the 0.025% cyCr group and 12.8 mM (range 9.2–15.2 mM) in the 0.1% cyCr group. There was no significant decrease in mean serum glucose concentration in any of the experimental groups compared with controls.

Intraperitoneal injections of cyCr were given in a dose calculated to correspond to the ingested dose of mice fed 0.1% cyCr (10 mg daily i.p.). This dose did not induce significant weight loss or other signs of toxicity when given intraperitoneally.

### $^{31}$P- and $^{1}$H-MRS

Typical in vivo $^{31}$P- and extract $^{1}$H-MR spectra are shown in Figure 3. Uptake of the substrates by the tumour was evident from the

© Cancer Research Campaign 1999

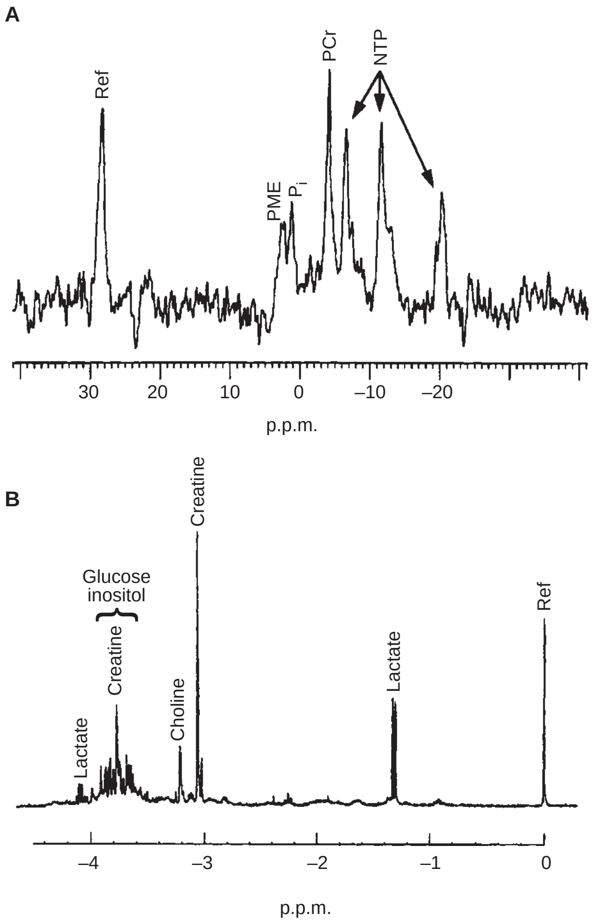

**Figure 3** Representative in vivo $^{31}$P-MR spectrum of a LS174T tumour from a mouse fed with 5% Cr (**A**). $^{1}$H-MR spectrum of tumour extract (**B**)

increase in PCr and PcyCr contents. The absolute levels of NTP and $P_i$ were not significantly different after 2 weeks of feeding with CK substrates, with the exception of the Cr 5% group. At this dietary concentration, $P_i$ concentrations were lower ($P < 0.05$). None of the experimental groups experienced a decrease in NTP/$P_i$, and reduction of $P_i$ caused a significant increase in this index only in mice fed with Cr 5% ($P < 0.05$). It can be seen from Table 1 that the PCr/$P_i$ ratio increased in the Cr 5% group ($P < 0.01$) and the PcyCr/$P_i$ ratio increased in both groups fed with cyCr ($P < 0.05$). Acidic intracellular pH values were observed in all of the experimental groups, as compared with controls ($P < 0.002$ and $P < 0.02$ with Cr and cyCr feeding respectively).

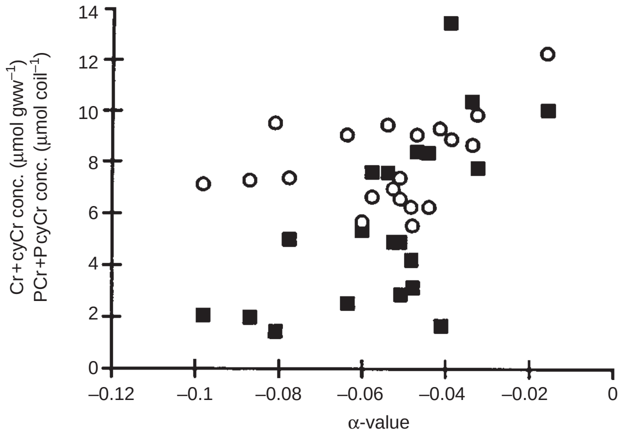

**Figure 4** Relationship between Cr+cyCr (■) and PCr + PcyCr (○) concentration and growth inhibition (α-value). Cr+cyCr was correlated to α ($r = 0.64$, $P = 0.002$). There was no significant correlation between PCr+PcyCr and α ($r = 0.25$, $P = 0.29$) (Spearman test)

Total PCr+Cr and PcyCr+cyCr contents in the tumours increased as a function of the dietary concentration (Table 1). In Cr-fed animals, intratumoral concentrations increased 2.5- and 5-fold at dietary concentrations of 2.5% and 5% Cr. Table 1 shows the levels of PCr and PcyCr detected in different tumours. It was not possible to distinguish PCr from PcyCr in the MR spectrum. The total increase in PCr+Cr was markedly larger than the increase in PCr concentrations. A similar disproportional increase in total vs phosphorylated substrates was observed in tumours of cyCr-fed animals.

There was a strong correlation between Cr+cyCr concentration and degree of growth inhibition (α-value) ($r = 0.64$, $P = 0.002$), whereas no correlation was found between growth inhibition and the concentration of phosphorylated compounds ($r = 0.25$, $P = 0.29$) (Figure 4).

Measurements of the magnetization transfer PCr to ATP are presented in Table 2. The reaction velocity was slightly decreased by Cr feeding (not significant). At the higher dose of cyCr, no exchange was detected, indicating that the major fraction of the $^{31}$P resonance was composed of PcyCr. At the lower cyCr dietary concentration, the rate constant of saturation transfer was markedly increased ($P < 0.01$ vs control), indicating the presence of PCr in these tumours. As the relative concentrations of PCr and PcyCr in these tumours were not known, the reaction velocity could not be estimated.

Mean steady-state lactate and alanine concentrations measured by $^{1}$H-MRS were 24.1 mmol g$^{-1}$ ww and 2.4 mmol g$^{-1}$ ww, respectively,

**Table 1** Metabolite concentrations, pH values and energy indices

| | Cr (µmol g$^{-1}$ ww) | cyCr (µmol g$^{-1}$ ww) | PCr+PcyCr (µmol coil$^{-1}$) | NTP (µmol coil$^{-1}$) | pH | Pxx/NTP | Pxx/$P_i$ | NTP/$P_i$ |
|---|---|---|---|---|---|---|---|---|
| Control | 2.0 ± 0.5 | – | 7.66 ± 1.38 | 4.96 ± 1.04 | 7.37 ± 0.07 | 1.8 ± 0.3 | 2.6 ± 0.7 | 1.4 ± 0.3 |
| Cr 2.5% | 4.8 ± 1.6[a] | – | 6.64 ± 1.92 | 3.44 ± 0.96 | 6.94 ± 0.12[a] | 2.2 ± 0.4 | 2.8 ± 0.7 | 1.3 ± 0.4 |
| Cr 5% | 10.3 ± 2.4[a] | – | 10.24 ± 1.6[a] | 4.24 ± 0.88 | 6.99 ± 0.12[a] | 2.8 ± 0.1[a] | 6.1 ± 1.1[a] | 2.2 ± 0.3[a] |
| cyCr 0.025% | 3.2 ± 0.9 | 1.2 ± 0.2[a] | 6.4 ± 1.60 | 3.6 ± 0.96 | 7.07 ± 0.10[a] | 2.2 ± 0.2 | 3.5 ± 0.3[a] | 1.6 ± 0.1 |
| cyCr 0.1% | 1.8 ± 0.4 | 6.2 ± 0.1[a] | 9.04 ± 2.32 | 3.92 ± 0.96 | 7.00 ± 0.05[a] | 2.6 ± 0.4[a] | 4.3 ± 0.3[a] | 1.7 ± 0.3 |

Values are given as means ± s.d. Pxx represents PCr and/or PcyCr. [a]$P < 0.05$ vs control.

© Cancer Research Campaign 1999

**Table 2** Pseudo-first-order rate constants (*k*) and reaction velocity (*v*) of phosphate transfer from PCr to NTP in the four experimental groups

| | *k* ($s^{-1}$) | *v* (mmol coil$^{-1}$ min$^{-1}$) |
|---|---|---|
| Control | 0.18 ± 0.03 | 1.04 ± 0.18 |
| Cr 2.5% | 0.15 ± 0.02 | 0.75 ± 0.2 |
| Cr 5% | 0.12 ± 0.02 | 0.92 ± 0.14 |
| cyCr 0.025% | 0.31 ± 0.04[a] | – |
| cyCr 0.1% | 0[a] | 0[a] |

[a]*P* < 0.05 vs control.

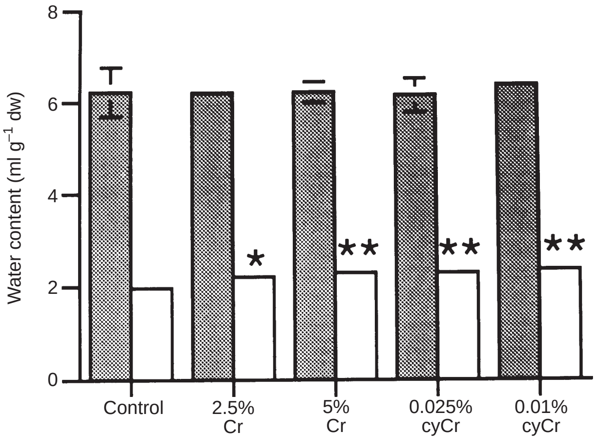

**Figure 5** Tissue water content in LS174T tumours (grey) and liver tissue (white) of nude mice. Experimental groups were compared with controls by a *t*-test. **P* < 0.05. ***P* < 0.01

in control tumours. Mean levels of lactate and alanine in the experimental groups did not differ significantly from controls (data not shown).

### CK activity

Total CK activity (PCr → Cr) in the tumours was 2.12 ± 0.23 μmol (mg protein)$^{-1}$ min$^{-1}$ and 270 ± 34 μmol g$^{-1}$ ww min$^{-1}$. Gel electrophoresis showed that 85 ± 5% of the activity was attributable to the BB dimer and 10 ± 3% to the MB dimer. The MM band was barely detectable. The presence of CK-mit was also apparent on concentrated gels but its partial activity was too low to be quantified. No significant changes in total CK activity and isoenzyme distribution were observed after feeding with Cr or cyCr.

### Tumour and hepatic water content

Total tumour water contents and liver water contents are presented in Figure 5. There was no significant difference in total water contents when tumours from Cr- and cyCr-fed mice were compared with controls. However, the total liver water contents were significantly higher than control values in all experimental groups.

### DISCUSSION

The data presented demonstrate a significant inhibitory effect of creatine and cyclocreatine on a human colon adenocarcinoma cell line implanted in nude mice. Growth inhibition was best correlated with intratumoral CK substrate concentrations, and was achieved at dietary Cr and cyCr doses as low as 2.5% and 0.1%, respectively, or daily doses of 10 mg cyCr i.p. In vivo, these agents did not decrease NTP/$P_i$ and did not cause excessive intratumoral water accumulation, as previously proposed for other cell lines in vitro. We propose that phosphorylation of Cr and cyCr by CK increases the intracellular accumulation of substrates, thus indirectly increasing their anti-tumoral effect.

### Energetic considerations

After 2 weeks of oral feeding with Cr or cyCr, the tumours showed no signs of energy deprivation measured as PCr+PcyCr/NTP and NTP/$P_i$. Cr and cyCr feeding increased the concentrations of the phosphorylated CK substrate, without significant changes in NTP and $P_i$ levels. It is evident that the increase in PCr+PcyCr/NTP was caused by enrichment of the tumours with CK substrates (Table 1). The phosphorylation potential ($[ATP][ADP]^{-1}$ $[P_i]^{-1}$) is important for regulation of oxidative metabolism and can be altered by changes in the relative concentrations of substrates and products in the CK equilibrium. At equilibrium, changes in the concentrations of Cr or cyCr and PCr or PcyCr will influence the concentrations of ATP, ADP and $H^+$ according to the equilibrium constant ($K_{eq}$):

$$K_{eq}\,(Cr) = [ATP] \times [Cr]/[ADP] \times [PCr] \times [H^+]$$

$$K_{eq}\,(cyCr) = [ATP] \times [cyCr]/[ADP] \times [PcyCr] \times [H^+]$$

In our study, the increase in Cr exceeds the increase in PCr, possibly leading to a decrease in ADP concentration, which will increase the phosphorylation potential and decrease the rate of oxidative metabolism. In contrast, the acidification of tumours may lower the ADP concentration (provided that all other concentrations are unchanged) and increase the oxidative phosphorylation rate. Thus, changes in oxidative phosphorylation/glycolysis rate may not be reflected in the MRS-detectable energetic ratios PCr+PcyCr/NTP and NTP/$P_i$. Unfortunately, calculation of the phosphorylation potential was not possible in these experiments.

The apparent increase in PCr+PcyCr/NTP ratio does not necessarily translate into an improved energetic state, and the tumours may actually be energy deprived as PcyCr is 30-fold more stable than PCr, and its turnover is 160 times less efficient in vitro (LoPresti et al, 1989). Consequently, the NTP/$P_i$ ratio may be better than the PCr+PcyCr/NTP ratio as an indicator of the energetic state of the tumour in the absence of ADP concentrations and calculations of phosphorylation potential. NTP/$P_i$ was not significantly affected in any of the experimental groups except for the group fed 5% Cr, in which its increase was the result of lower $P_i$ concentrations (Table 1). Growth inhibition does not seem to be caused by energy deficiency measured by MRS as NTP/$P_i$ or PCr+PcyCr/$P_i$, but other energetic effects not reflected in these ratios cannot be excluded.

It is evident from the present results that Cr and cyCr had a direct cytostatic effect on tumour cells, as previously demonstrated in vitro (Schiffenbauer et al, 1996). Growth inhibition was associated with tumour acidification, starting from relatively alkaline pH values in controls, characteristic of most untreated tumours (Griffith, 1991). The effect of Cr and cyCr may be mediated by lactate accumulation secondary to inhibition of oxidative phosphorylation. However, the low pH values were not accompanied by high intratumoral lactate concentrations. The observation that lactate concentrations were not raised further supports the concept

 © Cancer Research Campaign 1999

that energy metabolism was not impaired. In parallel, high plasma glucose levels indicate ample substrate delivery to the tumour, and the stable NTP/$P_i$ ratio suggests that the tumours were adequately perfused. This index was shown to be a sensitive detector of changes in blood flow in ex vivo mammary adenocarcinoma preparations; in this tumour NTP/$P_i$ was most sensitive to changes in carbon substrate supply and less to oxygen delivery (Eskey et al, 1993). The present experiments address directly only chronic effects of Cr and cyCr administration, via an oral route, in tumour-bearing mice. Our findings corroborate the results of Schiffenbauer et al (1996), who found no changes in NTP levels and a gradual increase in PcyCr during 12–20 h of acute cyCr administration in vitro.

The undetectable phosphate transfer rate between PCr and γ-NTP in the 0.1% cyCr group indicated that this tumour contained almost exclusively PcyCr. The turnover of phosphate between PcyCr and NTP is slower than the nuclear magnetic resonance (NMR)-detectable time scale for saturation transfer. It follows, from the increased rate of saturation transfer in the 0.025% cyCr group, that, whenever present, PCr was the donor/acceptor of high-energy phosphate. Although the kinetic properties of PcyCr may induce changes in intracellular environment, such as lowering ADP levels, its chronic administration did not decrease NTP/$P_i$ or total NTP concentration (Table 1). Chronic cyCr administration allowed the replenishment of $P_i$, as there were no signs of a decrease in $P_i$ concentration in any of the cyCr-treated groups.

### Growth inhibition by Cr and cyCr

In the present experiments, we found a close relationship between growth inhibition and concentration of Cr and cyCr (Figure 4 and Table 1) that was consistent with a direct toxic effect of these CK substrates. In contrast to the total substrate concentrations, there was a very poor correlation between growth inhibition and the steady-state intratumoral concentration of PCr and PcyCr (Figure 4). Thus, the concentration of substrate seems to be more important than the concentration of phosphorylated products for the growth-inhibitory effect of both compounds. Intracellular 'trapping' of Cr and cyCr due to phosphorylation may well occur and would explain the increased effect of cyCr in tumours with high CK activity and after transfection of cancer cells with the CK gene (Lillie et al, 1993). This possibility is further supported by the fact that there seems to be a steep dose–response curve in the dose range applied in the present experiments (Figure 1) and in experiments by other investigators (Lillie et al, 1993; Miller et al, 1993).

It is noteworthy that the total tumour concentrations of cyCr and Cr were comparable (6.2 ± 0.1 in the 0.1% cyCr group compared with 10.3 ± 2.4 in the 5% Cr group), despite a dietary Cr/cyCr ratio of 50:1 (Table 1). These numbers indicate a preferential uptake of cyCr into the tumour tissue. The role of a specific sodium-dependent transporter of Cr and cyCr (Guimbal et al, 1993; Sora et al, 1994) remains to be determined. This transport molecule may have a higher affinity for cyCr than Cr. Estimates indicate that 48–67% of Cr transport from interstitial fluid to the intracellular space is sodium dependent at $[Na^+]$ = 155 mM and [cyCr] = 5 mM (Schiffenbauer et al, 1996). At cyCr concentrations much greater than the estimated $K_m$ value of 0.54 mM, passive diffusion becomes more important. As cyCr is found, in this and other (Schiffenbauer et al, 1996) studies, to be active at a concentration of 5 mM, the co-transport of sodium and Cr/cyCr may be involved in the mechanism of action of these compounds. The in vivo growth-inhibitory effect was achieved at an oral dose of cyCr 5–10 times lower than in the studies by Lillie et al (1993) and Miller et al (1993), whereas the effective i.p. dose in this study (10 mg daily) was comparable to the i.p. dose used by others (Teicher et al, 1995; Schimmel et al, 1996). However, the intratumoral substrate concentration of 5.9 mM reached in the 0.1% cyCr group (Table 1) was very close to the reported in vitro growth-inhibitory levels of 7–20 mM (Martin et al, 1994*a*). The fact that growth inhibition was observed at similar concentrations in vitro and in vivo demonstrates that the effect of cyCr on these tumours are not caused by collapse of stromal components, e.g. tumour vasculature.

### Involvement of CK in growth

Schiffenbauer et al (1996) examined two different cell lines for cyCr content; the cyCr-sensitive rat glioma cell line C6 had a CK activity of 0.16 unit $mg^{-1}$ and a dramatic increase in intracellular PcyCr content during cyCr perfusion, whereas the CK activity of the less sensitive ovarian carcinoma line OC238 was tenfold lower and did not seem to accumulate either PcyCr or cyCr. These observations may be explained by slower phosphorylation in the OC238 cells (as a result of the lower CK activity), leading to a slower accumulation of the total amount of cyCr in these cells.

The CK activity of 2.1 units $mg^{-1}$ is one order of magnitude higher than that of the tumour line with the highest activity in the study by Schiffenbauer et al (1996), and exactly the same as the activity previously reported for the colon adenocarcinoma cell line Caco-2 (Lillie et al, 1993). The relatively high CK activity in LS174T may explain the very high sensitivity of these tumours to Cr and cyCr at very low doses. On the other hand, the CK activity will only influence the phosphorylation rate and not the intracellular concentration of PCr and PcyCr, provided that the system is in equilibrium. Thus, once the steady-state concentration of PCr/PcyCr is reached, the CK activity of the tumour cells may be less important than the given dose or the plasma concentration of substrate. It is unknown whether the Cr transporter is expressed in parallel or is independent of CK.

A recent study has shown that subcellular localization of the isoenzymes may be of importance for hepatocyte regeneration, as liver regeneration was impaired in transgenic mice with livers expressing CK-mit fed Cr and in mice with livers expressing CK-B fed cyCr (Askenasy and Koretsky, 1997). CK-B is the most abundant isoform in most tumours (DeLuca et al, 1981; Lillie et al, 1993) and fetal tissues (Hall and DeLuca, 1976). In LS174T, only cytosolic isoenzymes of CK were detected with an expression of ≈90% BB and ≈10% MB. The association of CK-B with growth, and the increased sensitivity of growing tissues containing this isoenzyme to cyCr, suggests that it may be worthwhile to investigate the isoenzyme distribution of tumours for two purposes: (1) to optimize the anti-tumour effect of Cr and cyCr and (2) to elucidate the interaction between enzymes and substrates in the mechanism of action of Cr and cyCr. The isozyme distribution in LS174T tumours (≈90% BB) is very close to the distribution in colon washings from normal subjects (Bereznitsky et al, 1982).

### Water accumulation

Neither Cr nor cyCr induced significant water accumulation in the tumour tissue in spite of significant growth inhibition (Figure 5). In contrast, both treatment modalities increased the total liver water content significantly, thus corroborating previous studies of

© Cancer Research Campaign 1999 

water accumulation in liver tissue after Cr and cyCr treatment (Askenasy and Koretsky, 1997). However, in that study, the induced hepatic oedema could not explain the inhibitory effect on liver growth. Correspondingly, we did not find evidence that the tumour growth-inhibitory effect is attributable to intratumoral water accumulation in vivo, although this mechanism has been suggested to be of importance for inhibition of in vitro tumour cell proliferation (Schiffenbauer et al, 1995). Similarly, the induction of oedema in itself could not explain the growth-inhibitory effect of cyCr on C6 or OC238 cells (Schiffenbauer et al, 1996).

### Systemic effects

Severe fasting and weight loss has previously been shown to inhibit tumour growth (Giovanella et al, 1982), but in the present experiments the lack of correlation between weight loss and growth inhibition indicated that weight loss was not the direct cause of growth inhibition. This is further corroborated by the observation that i.p. injections of cyCr did not induce weight loss, but did inhibit tumour growth (Figure 2), at a dose (10 mg i.p. daily) directly comparable to the amount of cyCr ingested by the mice fed 0.1% cyCr. Finally, because both colon tumours (Bartholomew and Schutt, 1971) and cyCr feeding cause mild hypoglycaemia (unpublished results), plasma glucose levels were measured. In the absence of significant differences in the experimental groups, growth inhibition cannot be attributed to the non-specific effects of starvation.

In this study, Cr and cyCr for feeding experiments were mixed in a viscous liquid diet instead of dry food. In previous experiments performed in our lab, we observed that the mice were able to avoid the cyCr crystals when mixed with powdered dry food. Consequently, the effective dose may be smaller than was assumed in the previous experiments by Lillie et al (1993) and Miller et al (1993). Also, the mouse strain may be of importance – nude mice seem to tolerate less cyCr than immunosufficient mice and there might be a difference in tolerance between different strains of nude mice.

It is possible that the effect of Cr and/or cyCr is mediated by acidification of the tumour tissue, but the exact mechanism of action of these compounds remains unknown. Further studies are warranted to elucidate the role of CK isozymes on the anti-tumour effect and to optimize treatment schedules for these drugs alone or combined with synergistically acting chemotherapeutic agents (Teicher et al, 1995).

### ACKNOWLEDGEMENTS

We thank Ms Sylvie Roberge for assistance with tumour transplantation and animal feeding. Dr Mildred Cohn is gratefully acknowledged for helpful comments on the manuscript. This work was supported by an Outstanding Investigator Grant (R35-CA-56591) from the National Cancer Institute (RKJ) and The Danish Medical Research Council (CAK). CAK is a post-doctoral fellow of The Michaelsen Foundation, Denmark.

### REFERENCES

Askenasy N and Koretsky AP (1997) Differential effects of creatine kinase isoenzymes and substrates on regeneration in livers of transgenic mice. *Am J Physiol* **273**: C741–C746

Bartholomew LG and Schutt AJ (1971) Systemic syndromes associated with neoplastic disease including cancer of the colon. *Cancer* **28**: 170–174

Bereznitsky S, Lobstein OE, Ko ST and Weinstock A (1982) Alterations of creatine kinase isoenzymes in colon washings from patients with colonic and rectal diseases. *Cancer* **50**: 1177–1180

Bergnes G, Yuan W, Khandekar VS, O'Keefe MM, Martin KJ, Teicher BA and Kaddurah-Daouk R (1996) Creatine and phosphocreatine analogs: anticancer activity and enzymatic analysis. *Oncol Res* **8**: 121–130

Carney DN, Zweig MH, Ihde DC, Cohen MH, Maduch RW and Gazdar AF (1984) Elevated serum creatine kinase BB levels in patients with small cell lung cancer. *Cancer Res* **44**: 5399–5403

DeLuca M, Hall N, Rice R and Kaplan NO (1981) Creatine kinase isozymes in human tumours. *Biochem Biophys Res Commun* **99**: 189–195

Eskey CJ, Koretsky AP, Domach MM and Jain RK (1993) Role of oxygen vs glucose in energy metabolism in a mammary carcinoma perfused ex vivo: direct measurement by $^{31}P$ NMR. *Proc Natl Acad Sci USA* **90**: 2646–2650

Giovanella BC, Shepard RC, Stehlin JS, Venditti JM and Abbott BJ (1982) Calorie restriction: effect on growth of human tumours heterotransplanted in nude mice. *J Natl Cancer Inst* **68**: 249–257

Griffith JR (1991) Are cancer cells acidic? *Br J Cancer* **64**: 425–427

Guimbal C and Kilimann MW (1993) A $Na^+$-dependent creatine transporter in rabbit brain, muscle, heart, and kidney. cDNA cloning and functional expression. *J Biol Chem* **268**: 8418–8421

Hall N and DeLuca M (1976) Electrophoretic separation and quantitation of creatine kinase isozymes. *Anal Biochem* **76**: 561–567

Kaddurah-Daouk R, Lillie JW, Daouk GH, Green MR, Kingston R and Schimmel P (1990) Induction of a cellular enzyme for energy metabolism by transforming domains of adenovirus E1a. *Mol Cell Biol* **10**: 1476–1483

Kristensen CA, Kristjansen PEG, Brünner N, Quistorff B and Spang-Thomsen M (1995) Growth inhibition in response to estrogen withdrawal and tamoxifen therapy of human breast cancer xenografts evaluated by in vivo $^{31}P$ magnetic resonance spectroscopy, creatine kinase activity, and apoptotic index. *Cancer Res* **55**: 4146–4150

Lillie JW, O'Keefe M, Valinski H, Hamlin HA, Varban ML and Kaddurah-Daouk R (1993) Cyclocreatine (1-carboxymethyl-2-iminoimidazolidine) inhibits growth of a broad spectrum of cancer cells derived from solid tumours. *Cancer Res* **53**: 3172–3178

LoPresti P and Cohn M (1989) Direct determination of creatine kinase equilibrium constants with creatine or cyclocreatine substrate. *Biochim Biophys Acta* **998**: 317–320

Martin KJ, Chen S-F, Clark GM, Degen D, Wajima M, Von Hoff DD and Kaddurah-Daouk R (1994*a*) Evaluation of creatine analogues as a new class of anticancer agents using freshly explanted human tumour cells. *J Natl Cancer Inst* **86**: 608–613

Martin KJ, Winslow ER and Kaddurah-Daouk R (1994b) Cell cycle studies of cyclocreatine, a new anticancer agent. *Cancer Res* **54**: 5160–5165

Martin KJ, Vassallo CD, Teicher BA and Kaddurah-Daouk R (1995) Microtubule stabilization and potentiation of taxol by the creatine analog cyclocreatine. *Anti-Cancer Drugs* **6**: 419–426

McAuliffe JJ, Perry SB, Brooks EE and Ingwall JS (1989) The kinetics of the creatine kinase reaction in neonatal rabbit heart: does the rate equation accurately describe the kinetics observed in the isolated perfused heart? *Prog Clin Biol Res* **315**: 581–592

Miller EE, Evans AE and Cohn M (1993) Inhibition of rate of tumour growth by creatine and cyclocreatine. *Proc Natl Acad Sci USA* **90**: 3304–3308

Neri B, Bartalucci S, Romano S and Cipriani A (1985) Evaluation of creatine kinase isoenzyme BB as a marker of neoplastic growth in Yoshida ascites hepatoma of the rat. *Anticancer Res* **5**: 533–536

Perry SB, McAuliffe JJ, Balschi JA, Hickey PR and Ingwall JS (1988) Velocity of the creatine kinase reaction in the neonatal rabbit heart: role of mitochondrial creatine kinase. *Biochemistry* **27**: 2165–2172

Schiffenbauer YS, Tempel C, Abramovitch R, Meir G and Neeman M (1995) Cyclocreatine accumulation leads to cellular swelling in C6 glioma multicellular spheroids: diffusion and one dimensional chemical shift nuclear magnetic resonance microscopy. *Cancer Res* **55**: 153–158

Schiffenbauer YS, Meir G, Cohn M and Neeman M (1996) Cyclocreatine transport and cytotoxicity in rat glioma and human ovarian carcinoma cells: $^{31}P$-NMR spectroscopy. *Am J Physiol* **270**: C160–C169

Schimmel L, Khandekar VS, Martin KJ, Riera T, Honan C, Shaw DG and Kaddurah-Daouk R (1996) The synthetic phosphagen cyclocreatine phosphate inhibits the growth of a broad spectrum of solid tumours. *Anticancer Res* **16**: 375–380

Shatton JB, Morris HP and Weinhouse S (1979) Creatine kinase activity and isozyme composition in normal tissues and neoplasms of rats and mice. *Cancer Res* **39**: 492–501

 © Cancer Research Campaign 1999

Sora I, Richman J, Santoro G, Wei H, Wang Y, Vanderah T, Horvath R, Nguyen M, Waite S, Roeske WR and Yamamura HI (1994) The cloning and expression of a human creatine transporter. *Biochem Biophys Res Commun* **204**: 419–427

Spang-Thomsen M, Nielsen A and Visfeldt J (1980) Growth curves of three human malignant tumours transplanted to nude mice. *Exp Cell Biol* **48**: 138–154

Teicher BA, Menon K, Northey D, Liu J, Kufe DW and Kaddurah-Daouk R (1995) Cyclocreatine in cancer chemotherapy. *Cancer Chemother Pharmacol* **35**: 411–416

Walliman T, Wyss M, Brdiczka D, Nicolay K and Eppenberger HM (1992) Intracellular compartmentation, structure and function of creatine kinase isoenzymes in tissues with high and fluctuating energy demands: the “phosphocreatine circuit” for cellular energy homeostasis. *Biochem J* **281**: 21–40

Zhao J, Schmieg FI, Simmons DT and Malloy GR (1994) Mouse p53 represses the rat brain creatine kinase gene but activates the rat muscle creatine kinase gene. *Mol Cell Biol* **14**: 8483–8492

*© Cancer Research Campaign 1999*
